# Mixed Matrix Membranes

**DOI:** 10.3390/membranes9110149

**Published:** 2019-11-10

**Authors:** Clara Casado-Coterillo

**Affiliations:** Department of Chemical and Biomolecular Engineering, E.T.S. Ingenieros Industriales y Telecomunicación, Universidad de Cantabria, Av. Los Castros, s/n, 39005 Santander, Cantabria, Spain; casadoc@unican.es; Tel.: +34-942-206777

**Keywords:** membrane fabrication, membrane modification, flat-sheet membrane, characterization techniques, hollow fiber membrane, filler dispersion, compatibility, gas separation, ion exchange capacity, water vapor

## Abstract

In recent decades, mixed matrix membranes (MMMs) have attracted considerable interest in research laboratories worldwide, motivated by the gap between the growing interest in developing novel mixed matrix membranes by various research groups and the lack of large-scale implementation. This Special Issue contains six publications dealing with the current opportunities and challenges of mixed matrix membranes development and applications as solutions for the environmental and health challenges of 21st century society.

## 1. Introduction

This Special Issue, entitled “Mixed Matrix Membranes”, was motivated by the observed gap between the growing interest of research laboratories in developing novel mixed matrix membranes (MMMs) and the lack of large-scale implementation. MMMs, consisting of the mixing of innovative fillers and processable polymer matrices, may fill in this gap for conventional membranes to address industrial process intensifications challenges [1]. The papers compiled within this Special issue can be read as single chapters of a global story orientated toward the advancement of mixed matrix membranes and novel materials in membrane technology in response to some technical challenges faced by chemical industries and society, from CO_2_ capture and utilization to tissue engineering applications in biomedicine. They are all connected through important issues regarding fabrication, such as compatibility and adhesion, the effect of porous and non-porous fillers on the polymer matrices, types of additives/fillers (zeolites, ionic liquids, ion-exchange materials, layered porous materials, metal organic frameworks (MOFs), etc.), and characterization (e.g., chemical, structural, morphological, electrical, compositional, mechanical and topographical properties, as well as membrane transport and separation). 

## 2. Highlights of the Special Issue

The papers included in this special issue direct the developments in MMMs to some of the major challenges faced by society in the 21st century, mainly CO_2_ separation from other gases as a way in which to tackle climate change, and biomedical applications. One of the most important aspects is thus the selection of the appropriate material for both the matrix and dispersed phases to eliminate non-ideal morphologies created at their interfaces [1]. With these aims, several kinds of membranes have been addressed, as will be presented in the following paragraphs.

### 2.1. Mixed Matrix Membranes with Porous Fillers

The Special Issue opens with a review presenting a complete synopsis of the inherent capacities of several porous nanofillers, distinguishing between two-dimensional (2D) and three-dimensional (3D) shaped fillers [2] for CO_2_ separation from other gases. Gas permeation performances of selected hybrids with 3D fillers and porous nanosheets have been summarized and discussed with respect to each type and the effects of their embedment in polymers to make mixed matrix membranes for the separation of CO_2_ from other gases [3]. The particular challenge of achieving an intimate adhesion between fillers and polymer matrices to avoid the presence of defects and assure a correct synergy of the new MMM material is addressed by the studies of metal organic frameworks (MOFs) [4], and porous organic frameworks (POFs) [5,6], in consideration of their organic nature and high CO_2_ uptake properties. The oldest studied MMMs with porous fillers and glassy polymers for gas separation are zeolite–polymer membranes. The additional porosity provide additional transport mechanisms that account for their non-ideal performance [7]. The prediction of the mixed matrix membrane permeability and selectivity has been explored by different morphological models that have been thoroughly reviewed [8]. The feature paper contained in this Special Issue compares several of those models regarding the effect of filler type and topology on CO_2_ and N_2_ permeability using zeolites of different topologies (CHA, RHO, and LTA) and identical Si/Al compositional ratio, embedded in a high permeability glassy polymer, poly(trimethylsilyl-1-propyne) (PTMSP), as a function of temperature, zeolite loading, and topology [9]. The evolution of temperature and its influence on non-idealities, such as membrane rigidification, zeolite–polymer compatibility, and sieve pore blockage, allow prediction of the structure-performance relationship for further membrane development for the first time [10].

The recent advances in the synthesis and improvements of 2D and 3D porous nanophases have driven continuous research within the development of MMMs for gas separation purposes. In particular, the possibility of tuning the pore diameter to a gas-sieving level and the CO_2_-philicity of the pore cavity has the potential to facilitate the simultaneous enhancement of the solubility and diffusivity coefficient of carbon dioxide and reduced CO_2_ plasticization when high pressures are necessary [11,12]. Therefore, CO_2_ permeability and selectivity can be expected to benefit from these features, leading to a shift in the separation performance towards the upper right corner of the Robeson plot as a function also of the rubbery or glassy nature of the polymer matrix [13]. 

2D porous fillers offer a high surface area to volume ratio that provides higher contact between the filler and the polymer matrix in comparison with other filler morphologies [14]. This may result in the development of new applications, such as those explored by Sanchez-Gonzalez et al. [15] in this Special Issue. Their paper aims at elucidating the applicability of poly(caprolactone) (PCL) and reduced graphene oxide (rGO) MMMs as scaffolds for in vitro neural regeneration, by correlating the morphological, chemical, and differential scanning calorimetry (DSC) results with the membrane performance under simulated in vitro culture conditions (phosphate buffer solution (PBS) at 37 °C) for 1 year. The high internal porosity of the membranes facilitated water permeation and resulted in an accelerated hydrolytic degradation and molecular weight reduction. The presence of the rGO nanoplatelets caused the pH to be barely affected, while accelerating the loss of mechanical stability of the membranes. However, it is envisioned that the gradual degradation of the PCL/rGO membranes could facilitate cells infiltration, interconnectivity, and tissue formation. The relationship between structure and function seems again highly important in the opening up of novel applications for MMMs.

### 2.2. Mixed Matrix Membranes Filled with Nonporous Fillers

Membranes must offer a high CO_2_ permeability in order to compete with conventional membranes or other separation processes in CO_2_ capture and climate change mitigation processes [16]. Organic–inorganic nanocomposite membranes resulting from the in situ generation of inorganic nanoparticles in the polymer matrix can offer much higher gas permeabilities with similar selectivities than MMMs prepared by dispersion of inorganic fillers in the polymer matrix [17]. In this Special Issue, Guerrero et al. [18] present two differently functionalized types of polyhedral oligomeric silsesquioxanes (POSS^®^) nanoparticles as additives for nanocomposite membranes for CO_2_ separation. Composite membranes were produced by casting a polyvinyl alcohol (PVA) layer, containing the functionalized POSS^®^ nanoparticles, on a polysulfone (PSf) porous support. The compatibility between the nanoparticles and the polymer was observed by FTIR. Differential scanning calorimetry (DSC) and dynamic mechanical analysis (DMA) show an increment of the crystalline regions affected by the conformation of the polymer chains, decreasing the gas separation performance. Moreover, these nanocomposite membranes did not show separation according to a facilitated transport mechanism, as might be expected based on their functionalized amino-groups; thus, solution-diffusion was the main mechanism responsible for the transport phenomena [19].

Tuning the polymer free volume available for transport by disrupting the polymer chain packing with nanosized particles also has an effect on the gas permeation and stability of highly permeable rigid polymers [20]. Khan et al. [21] proposed here yet another nanofiller, potassium dodecahydrododecaborate (K_2_B_12_H_12_)—a polynuclear borane with potential in materials science and biomedicine—as a new filler to be added to the rigid structure of PIM-1 in order to improve its gas permeation properties and robustness [22]. Although the permeability performance of the prepared MMMs mainly depended on the addition of nanofillers rather than the effect of interfacial zone and the O_2_/N_2_ separation factor was almost constant for all the membranes, overall increases in permeability and diffusivity were observed for all tested gases coupled with the reduction in gas pair selectivity. 

### 2.3. Mixed Matrix Membranes Filled with Ionic Liquids

The search for a good adhesion between polymers and fillers has also been directed to ionic liquids (ILs). ILs have been thoroughly explored in the last few decades as an alternative form of solvent to amines in CO_2_ separation processes in supported ionic liquid membranes because of several advantages, such as negligible vapor pressure [16]. The combination of ionic liquids into a polymer matrix is an approach to enhance the mechanical stability of the separation process by avoiding working with a fluid phase [23,24]. This Special Issue presents an experimental study exploring the potential of supported ionic liquid membranes (SILMs) prepared by infiltration of protic imidazolium ionic liquids (ILs) into randomly nanoporous polybenzimidazole (PBI) membranes for CH_4_/N_2_ separation [25]. The polymerization, monitored by Fourier transform infrared (FTIR) spectroscopy, and the concentration of the protic entities in the membranes evaluated by thermogravimetric analysis (TGA) were correlated to the gas permeability values of N_2_ and CH_4_ at 313 K, 333 K, and 363 K in terms of the preferential cavity formation and favorable solvation of methane in the apolar domains of the protic ionic network. The transport mechanism of the as-prepared SILMs is solubility-dominated at high temperature, which can be compared with MMMs of similar components [26].

## 3. Final Remarks

Overall, the editor is convinced that mixed matrix membranes have a lot more to contribute than what has already been demonstrated worldwide. It is hoped that readers enjoy this Special Issue and gain inspiration from it for their own work. In the end, technological changes are the fruit of ideas planted as seeds in researchers’ minds: the more that individual minds are connected to each other, the higher the probability of creating originality. In this sense, this Special Issue represents a small attempt to increase the connectivity among interested minds, regarding the contributions to solve technological challenges in mixed matrix membrane development, and it shows the possibilities of synergies that the combination of compatible fillers and polymers can offer to environmental and health issues faced by society in the 21st century.
